# Hounsfield Unit for Assessing Bone Mineral Density Distribution Within Cervical Vertebrae and Its Correlation With the Intervertebral Disc Degeneration

**DOI:** 10.3389/fendo.2022.920167

**Published:** 2022-07-08

**Authors:** Xiao Liang, Qingtao Liu, Jiaxin Xu, Wenyuan Ding, Hui Wang

**Affiliations:** ^1^ Department of Spinal Surgery, The Third Hospital of Hebei Medical University, Shijiazhuang, China; ^2^ Hebei Provincial Key Laboratory of Orthopedic Biomechanics, The Third Hospital of Hebei Medical University, Shijiazhuang, China

**Keywords:** Hounsfield unit, bone mineral density distribution, intervertebral disc degeneration, cervical vertebrae, osteoporosis

## Abstract

**Study Design:**

Retrospective radiological analysis.

**Objective:**

To assess bone mineral mass distribution within cervical vertebrae based on Hounsfield unit (HU) measurement, and explore its correlation with intervertebral disc degeneration.

**Method:**

Three hundred and twenty-four patients with degenerative cervical spine disease were retrospectively reviewed and divided into six groups according to age. HU measurement of the whole vertebrae from C3 through C7 was obtained, then HU measurement within upper and lower part of the vertebrae on sagittal plane were obtained from C3 through C7. Disc degeneration on MRI was graded from I to V using the Pfirrmann classification.

**Results:**

There was a significant difference in the HU value from C3 to C7 among Group II to Group VI, the HU value presented consistently decreasing trend from young patients to old patients. In C6 and C7 vertebrae, there were significant differences in HU values between upper and lower parts of the vertebrae. More importantly. In all groups, HU values were highest in the upper part of the C4 vertebrae and then gradually decreased towards C3 and C7. HU value of both upper and lower vertebrae presented decreasing trend along with the aggravation of the disc degeneration.

**Conclusion:**

HU values are not typically consistent throughout all levels of the cervical spine and the distribution within the vertebrae is not homogeneous. Decreased vertebral BMD and vertebral osteoporosis may trigger or exacerbate the adjacent intervertebral disc degeneration.

## Introduction

Osteoporosis is a systemic bone disease caused by a decrease in bone density and quality, destruction of bone microstructure and increased bone fragility, which puts patients at increased risk of fracture. It is most common in the elderly and postmenopausal women. The bone mineral density(BMD) of cancellous bone is more susceptible to osteoporosis than that of cortical bone (1). Low BMD increases the difficulty of spinal surgery and increases the incidence of complications because the pullout strength of pedicle screws is highly correlated with BMD (1–3).

Dual-energy X-ray absorptiometry (DEXA) is the most commonly used method to determine BMD, and the World Health Organization (WHO) defines a T-scores of less than -2.5 as the gold standard for the diagnosis of osteoporosis. However, DEXA has limitations in assessing the condition of the vertebrae. It cannot distinguish between cortical and cancellous bone, and it also affected by vertebral osteophytes and calcifications that occur in the surrounding vascular wall. This may bias the assessment of localised BMD of the vertebrae in the elderly (4–6). Therefore, it is clinically important to accurately determine the BMD of the vertebral body, especially in cancellous bone. The Hounsfield Unit (HU) is a density metric on computed tomography (CT). Previous studies have shown that the HU values of vertebrae obtained on CT can be representative of the BMD of the vertebral body (5–11). It has the advantage that the average HU value of the vertebral region of interest can be measured in CT, which can improve the accuracy of the measurement. Therefore, CT can be used as a simple and effective way to evaluate BMD of the spine. What’s more, a correlation between vertebrae BMD and degenerative diseases of lumbar spine has been established (12). Whether the same is true in the cervical spine is not known.

The objectives of this study were: firstly, to explore the characteristics of cancellous BMD distribution in cervical vertebrae based on HU measurements; and secondly, to investigate the correlation between BMD in cervical vertebrae and adjacent intervertebral disc degeneration.

## Methods

### Patients

Inclusion criteria: 1. Degenerative cervical spine disease patients. 2. Full cervical spine Postero-Anterior (P/A) X-ray, cervical CT was available for HU measurement, cervical MRI was available for disc degeneration evaluation. 3. Age between 21 and 80 years old. Exclusion criteria: 1. Ossification of the posterior longitudinal ligament, spondylolisthesis, spinyal deformity, developmental spinal stenosis, tuberculosis, tumors, fractures, inflammation. 2. Coronal and sagittal imbalance, previous cervical surgery. 3. Addiction to alcohol and tobacco (smoking more than 2 cigarettes/day, drinking more than 50 mL/day).

By retrieving the medical records from January 2017 to December 2020 in our hospital, 324 patients who met both the inclusion and exclusion criteria were retrospectively reviewed, they were divided into six groups according to age: Group I(21-30 years old), Group II (31-40 years old), Group III (41-50 years old), Group IV (51-60 years old), Group V (61-70 years old), Group VI (71-80 years old).

### Data Collection and Assessment

Patient demographics including age and gender were recorded. All radiographic parameters were measured by two independent observers (first and second author). The C2–C7 Cobb angle was defined as the angle between the C2 inferior endplate and the C7 superior endplate.

**Figure 1:**
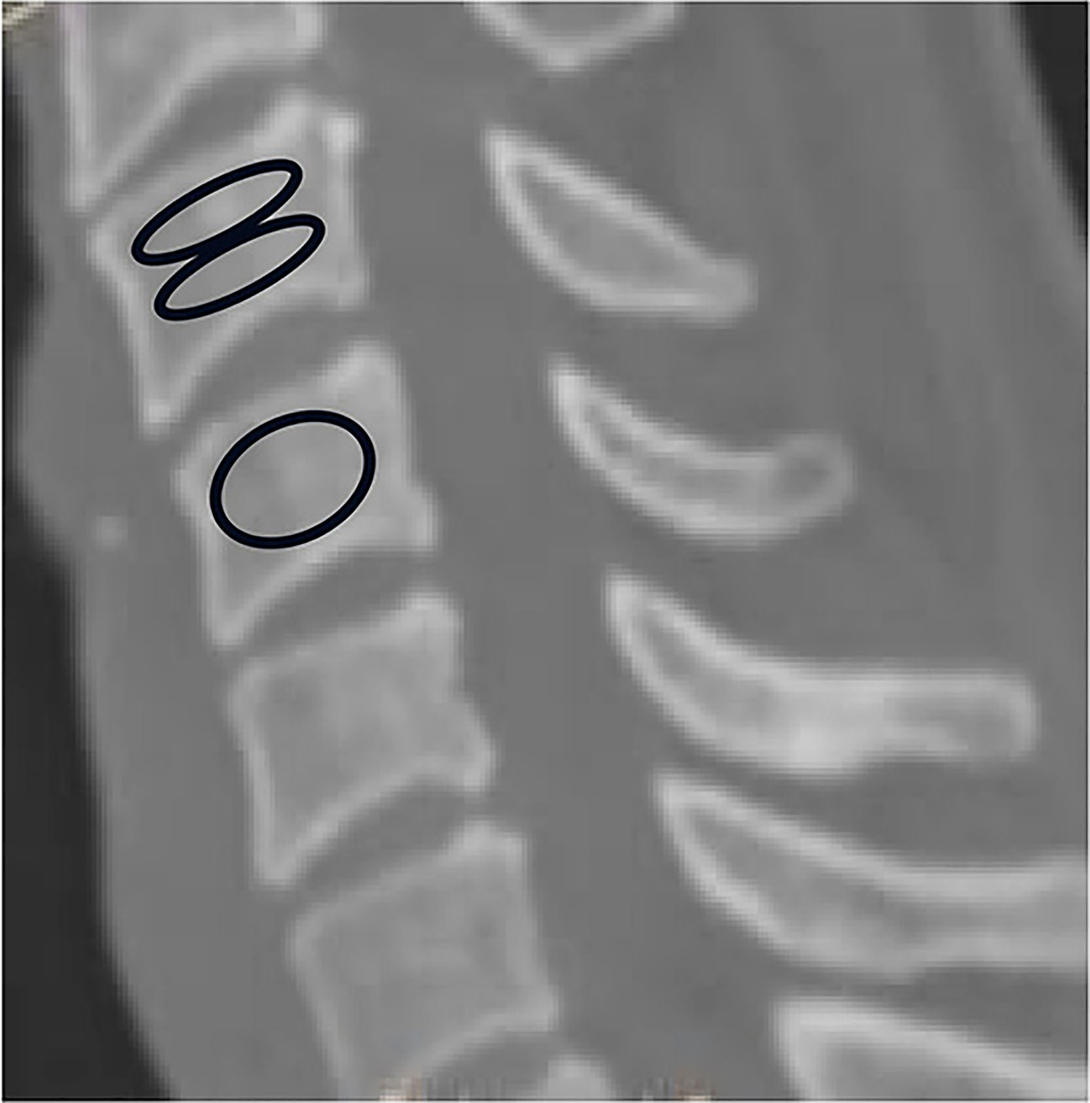
Computed tomography scan illustrating the method of determining the HU value with use of an elliptical region of interest.

The HU measurement for each vertebra was obtained by using a protocol described similar to Schreiber on CT examination (11). All subjects were scanned with a 64 slice multi-detector CT scanner (Siemens Sensation 64, Erlangen, Germany) according to the following parameters:slice thickness 1.5 mm, distance 1.5 mm, tube voltage 120 kV. Two-dimensional reconstructions were obtained in the sagittal plane. HU measurements were obtained from PACS (Picture Archiving and Communication Systems) Imaging System for C3 to C7. The region of interest for whole vertebraes were measured on Mid-sagittal images of the vertebrae, the largest possible elliptical region of interest was drawn, excluding the cortical margins to prevent volume averaging. Then HU measurement within the different region on mid-sagittal images of the vertebrae were obtained separately from C3 through C7: immediately inferior to the upper endplate (upper 1/2 part),and superior to the lower endplate (lower 1/2 part). Basivertebral vein foramen should not be included in the measurement of the whole vertebrae and different region of the vertebrae (**Figure 1**).

**Figure 2:**
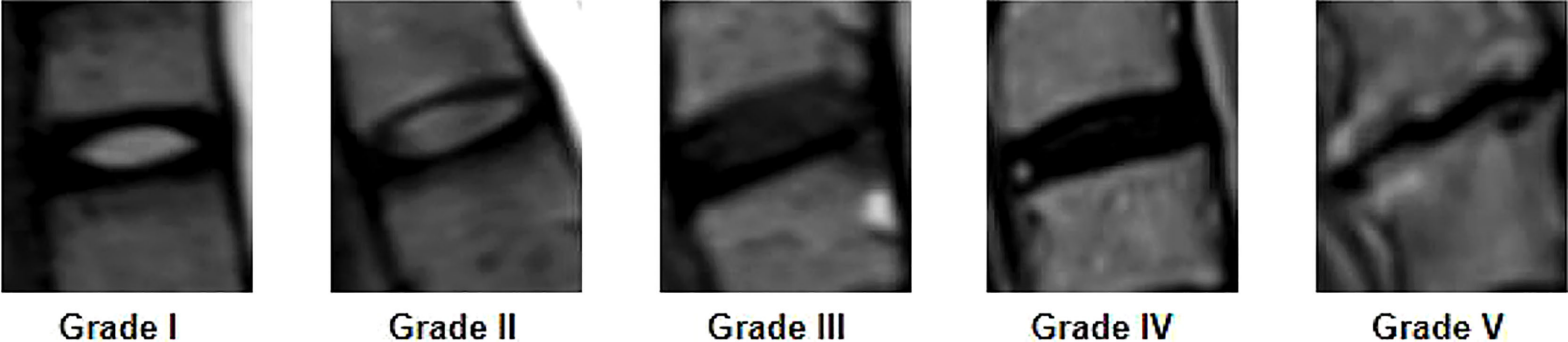
Cervical disc rating on MRI by using the Pfirrmann classification from grade I to grade V.

Disc degeneration on MRI was rated from grade I to V by using the Pfirrmann classification. Grade I: the structure of the disc is homogeneous, with bright hyperintense white signal intensity and normal disc height. Grade II: the structure of the disc is inhomogeneous, with the hyperintense white signal. Grade III: the structure of the disc is inhomogeneous, with an intermittent gray signal intensity. Grade IV: the structure of the disc is inhomogeneous, with a hypointense dark gray signal intensity. Grade V: the structure of the disc is inhomogeneous, with a hypointense black signal intensity. If the two independent observers presented different Pfirrmann Grade evaluation in the same disc, then the third author was invited to make the final decision (**Figure 2**).

### Statistical Analysis

Data were analyzed using Statistical Product and Service Solutions software (version 26; SPSS, Chicago, IL). Continuous variables were recorded as mean ± standard deviation, and categorical variables were expressed as frequency or percentages. Analysis of Variance (ANOVA) was used to compare the HU value among multi-subgroups. The rank sum test is used to compare samples with uneven variances. Paired samples t-test was used to compare the intravertebrae HU values. The association between the Pfirrmann grade of the disc and the HU values of the adjacent vertebrae were assessed using Spearman’s correlation coefficient. The statistical significance was set at *p* < 0.05.

## Results

### Characteristics of the Subjects

A total of 324 patients were included in this research, of whom 162 (50%) were male and 162 (50%) were female, with an average of 53.3 ± 12.7 years old. The minimum age 26 years old and the maximum age 79 years old. The C2–C7 Cobb angle was statistically different among subgroups(F=10.229 P<0.01), it presented consistently increasing trend from young (21-30 years old) subgroup to old (71-80 years old) subgroup (**Table 1**).

**Table 1:** Comparison of general data and Cobb among the six different age subgroups.

	Group I:21-30years	Group II:31-40years	Group III:41-50years	Group IV:51-60years	Group V:61-70years	Group VI:71-80years	statistic	*p*
Age	28.9 ± 1.5	36.5 ± 2.7	46.4 ± 2.5	55.1 ± 2.7	64.9 ± 2.9	73.6 ± 2.3	1422.3	<0.01
Cobb	-1.8 ± 10.8	3.7 ± 12.5	8.1 ± 10.4	12.1 ± 10.7	14.6 ± 11.9	15.3 ± 10.9	10.229	<0.01
Cases	8	58	72	84	62	40	–	–
man	4	29	36	42	31	20	–	–
woman	4	29	36	42	31	20	–	–

### HU Value Within the Cervical Vertebrae Among Six Different Age Subgroups

There was a significant difference in the HU value from C3 to C7 among Group II to Group VI, the HU value presented consistently decreasing trend from Group I to Group V (**Table 2**).

**Table 2:** HU value of cervical vertebrae from C3 to C7 in all of the six different age subgroups.

	Group I: 21-30years	Group II: 31-40years	Group III: 41-50years	Group IV: 51-60years	Group V: 61-70years	Group VI: 71-80years	statistics	*p*	Mean value
C3	397.8. ± 94.8	377.7 ± 68.4	364.6 ± 68.7	332.5 ± 86.8	316.3 ± 81.4	325.2 ± 82.1	6.232	<0.01	345.3 ± 81.6
C4	416.7 ± 59.4	400.1 ± 74.8	377.4 ± 72.1	342.5 ± 78.4	323.2 ± 86.1	330.8 ± 99.1	8.718	<0.01	357.2 ± 85.1
C5	389.0 ± 64.2	385.1 ± 74.8	359.5 ± 72.4	331.8 ± 83.3	298.8 ± 82.2	305.0 ± 86.5	10.049	<0.01	339.3 ± 85.7
C6	368.0 ± 56.2	350.0 ± 66.7	315.0 ± 74.3	286.8 ± 74.0	261.8 ± 78.0	266.32 ± 82.6	12.598	<0.01	299.1 ± 80.8
C7	325.9 ± 57.3	307.1 ± 61.8	284.1 ± 60.0	257.2 ± 58.4	234.7 ± 70.8	235.4 ± 64.0	13.012	<0.01	266.8 ± 68.1
statistics	2.083	15.561	78.588	19.079	13.791	9.482	–	–	258.836
*p*	0.104	<0.01	<0.01	<0.01	<0.01	<0.01	–	–	<0.01

A two-by-two comparison between the individual vertebrae of the different subgroups showed statistically significant differences between Group I-III and Group IV-VI, but no statistically significant differences between the individual vertebrae of Group V and Group VI (**Table 3**).

**Table 3:** P values for comparison of HU values of respective vertebrae among different subgroups.

Sections	Group	Group I	Group II	Group III	Group IV	Group V
C3	Group II	0.497				
	Group III	0.257	0.345			
	Group IV	0.025*	0.001*	0.011*		
	Group V	0.006*	0.000*	0.000*	0.219	
	Group VI	0.017*	0.001*	0.011*	0.629	0.576
C4	Group II	0.585				
	Group III	0.191	0.110			
	Group IV	0.013*	0.000*	0.007*		
	Group V	0.002*	0.000*	0.000*	0.152	
	Group VI	0.006*	0.000*	0.004*	0.447	0.643
C5	Group II	0.898				
	Group III	0.325	0.072			
	Group IV	0.055	0.000*	0.032*		
	Group V	0.003*	0.000*	0.000*	0.015*	
	Group VI	0.007*	0.000*	0.001*	0.084	0.702
C6	Group II	0.522				
	Group III	0.057	0.008*			
	Group IV	0.003*	0.000*	0.019*		
	Group V	0.000*	0.000*	0.000*	0.045*	
	Group VI	0.000*	0.000*	0.001*	0.154	0.762
C7	Group II	0.425				
	Group III	0.074	0.038*			
	Group IV	0.003*	0.000*	0.008*		
	Group V	0.000*	0.000*	0.000*	0.033*	
	Group VI	0.000*	0.000*	0.000*	0.071	0.953

(*p<0.05).

**Figure 3:**
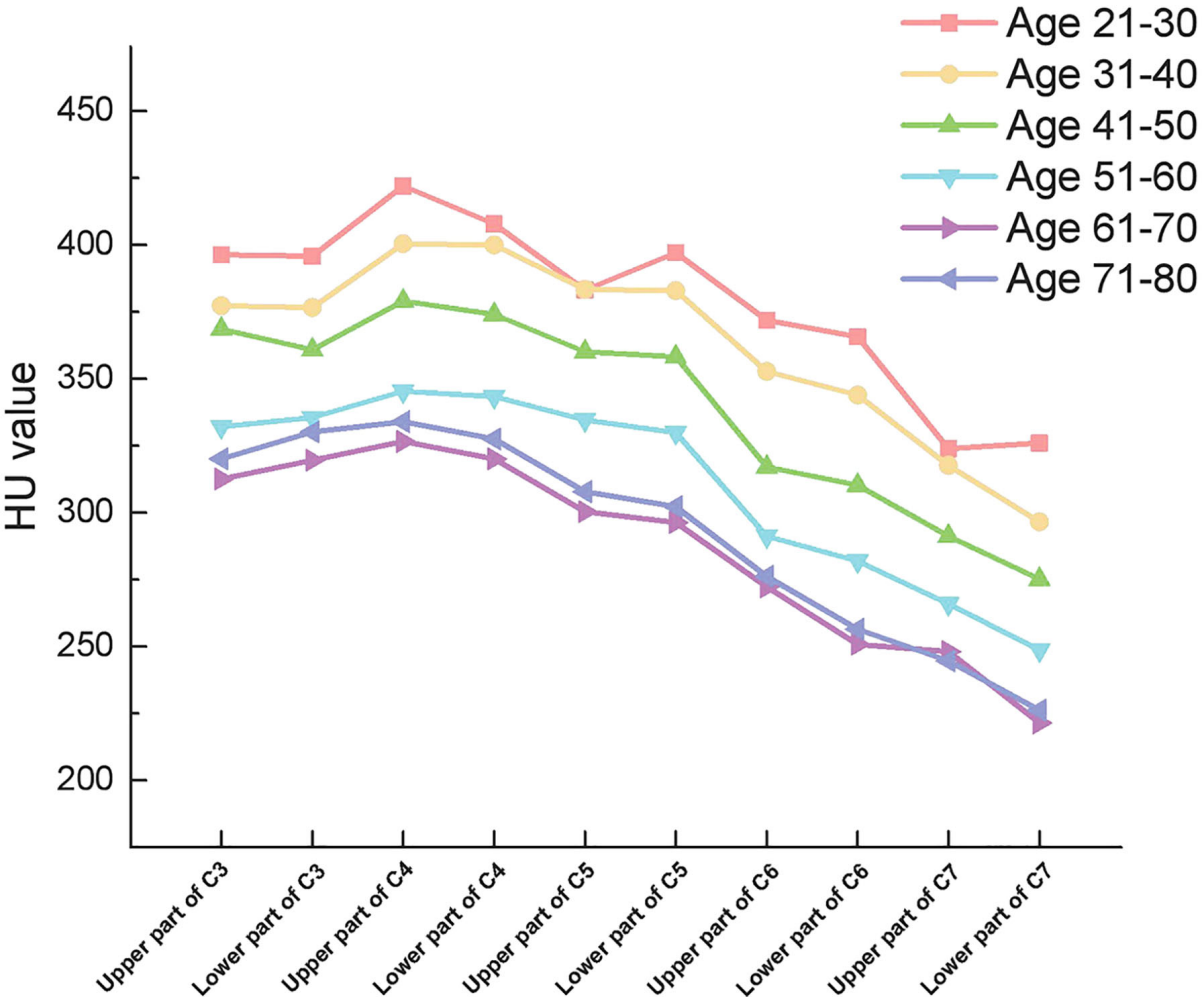
Outline of mean HU values of vertebrae from upper part of C3 to lower part of C7 in different subgroups.

From C3 to C5, there were no significant differences in HU values between the upper and lower parts of the vertebrae. However, there were significant differences in HU values between the upper and lower parts of the vertebrae in C6 and C7 vertebrae, respectively (**Table 4**). More importantly. In all groups, HU values were highest in the upper part of the C4 vertebrae and then gradually decreased towards C3 and C7. (**Figure 3**)

**Table 4:** HU value distribution within cervical vertebrae from C3 to C7.

	Upper part of vertebrae	Lower part of vertebrae	statistics	*p*
C3	344.7 ± 80.1	346.3 ± 83.6	-0.652	0.515
C4	359.6 ± 85.7	355.5 ± 89.7	1.855	0.064
C5	340.3 ± 85.3	337.5 ± 89.8	1.236	0.217
C6	304.5 ± 80.3	292.3 ± 85.4	5.657	<0.01
C7	276.2 ± 70.4	257.1 ± 69.9	9.604	<0.01

### Correlation of Cervical Vertebrae HU Value With the Intervertebral Disc Degeneration

Among the 1296 discs in the 324 patients, 1 disc was classified as Pfirrmann Grade I, 7 discs were classified as Pfirrmann Grade II, 524 discs were classified as Pfirrmann Grade III, 576 discs were classified as Pfirrmann Grade IV, 188 discs were classified as Pfirrmann Grade V. The HU value of both the upper vertebrae and lower vertebrae of the disc presented consistently decreasing trend along with the aggravation of the disc degeneration. (**Table 5**)

**Table 5:** Comparison of vertebrae HU value adjacent to discs of different Pfirrmann Grade. (* means significant difference was detected when compared to Grade II, & means significant difference was detected when compared to Grade III, # means significant difference was detected when compared to Grade IV).

Pfirrmann Grade	Cases	upper vertebrae	lower vertebrae
Grade I	1	–	–
Grade II	7	398.1 ± 74.6 #	351.4 ± 90.9
Grade III	524	348.9 ± 88.1 #	330.5 ± 91.4 #
Grade IV	576	328.8 ± 81.7 *&	311.6 ± 82.3 &
Grade V	188	314.6 ± 87.5 *&;#	285.4 ± 83.7 *&#
statistics		10.533	13.715
*p*		0.000	0.000

**Figure 4:**
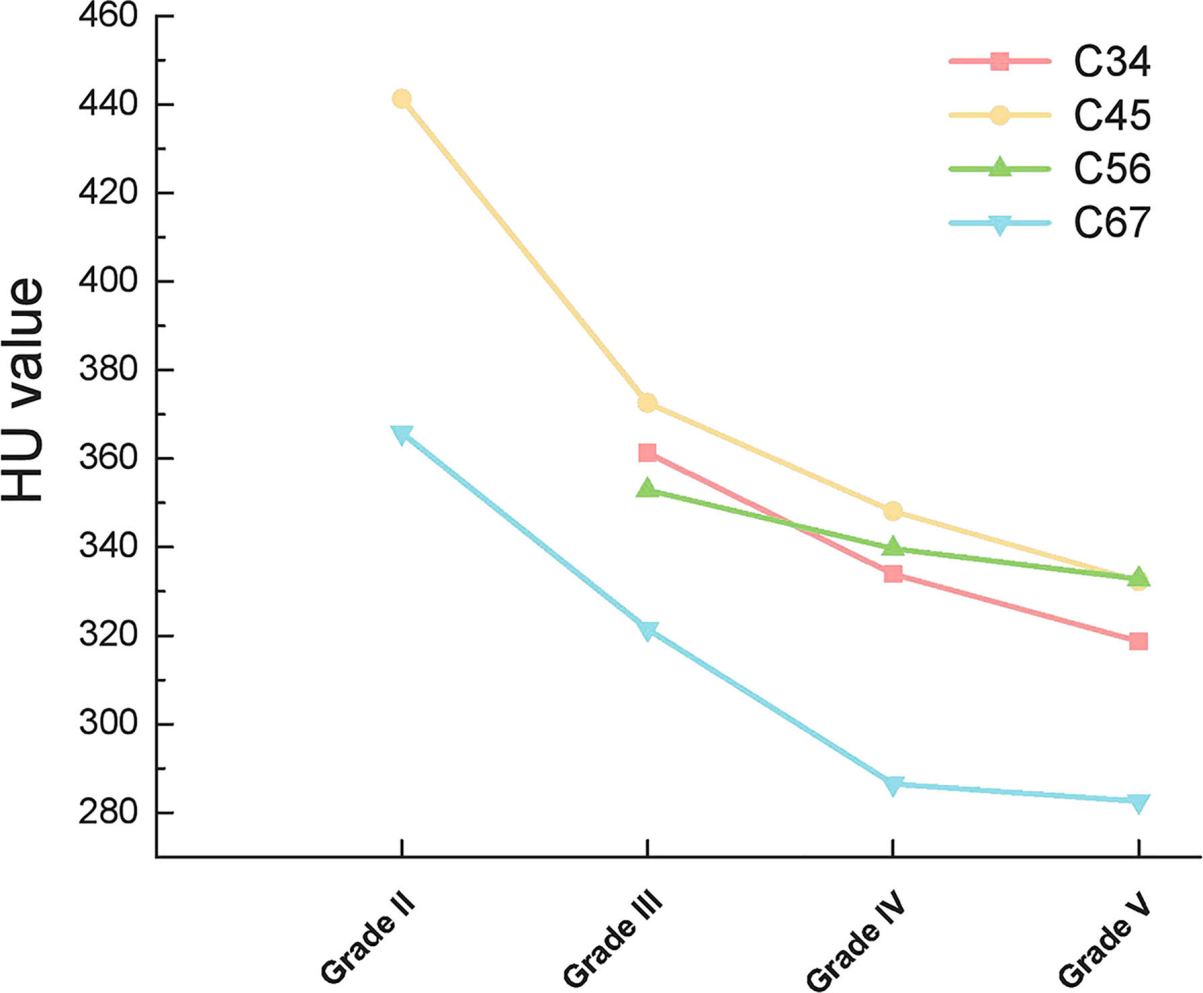
Outline of mean HU values of the upper vertebrae of different discs.

**Figure 5:**
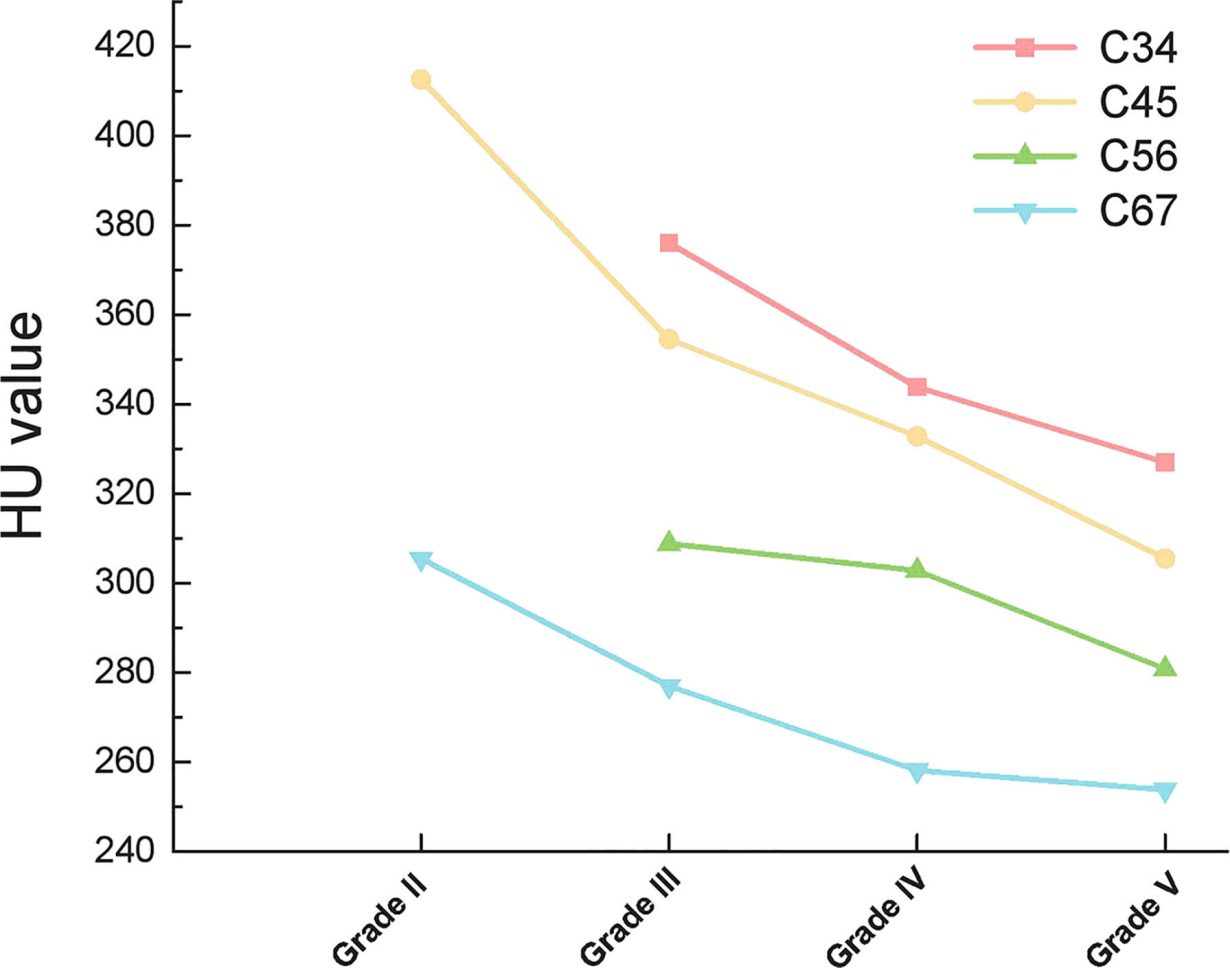
Outline of mean HU values of the lower vertebrae of different discs.

When compared according to the different disc segments, the HU value of both the upper vertebrae and lower vertebrae of the disc still presented consistently decreasing trend along with the aggravation of the disc degeneration (**Figures 4**, **5**).

Correlation analysis between different disc Pfirrmann grades and HU values of adjacent vertebrae demonstrated that there was a negative correlation between disc degeneration grades and HU values of adjacent vertebrae, p < 0.01 (**Table 6**).

**Table 6:** Correlation between different disc Pfirrmann grades and HU values of adjacent vertebrae.

	Correlation Coefficient	p
upper vertebrae	-0.141	<0.01
lower vertebrae	-0.150	<0.01

## Discussion

### The Effect of Age on HU Values of Cervical Vertebrae

With the ageing of the body, the functions of all the organs in the body deteriorate. The same trend applies to the skeletal system. Studies have shown that human cancellous bone loss with age begins in early adulthood (13). In our study, we stratified our patients by age and also confirmed that the HU values of the cancellous bone of the cervical vertebrae declined progressively with age. We consider that there may be several reasons for this. Firstly, older people tend to spend less time outdoors compared to younger people, which may affect calcium metabolism. Secondly, the dynamic balance between osteoblasts and osteoclasts may change with age, especially in postmenopausal women, leading to a decrease in cancellous bone density. In addition, both previous studies and our data suggest that the Cobb of the cervical spine gradually increases with age (14, 15), which can produce changes in cervical force lines that may affect the BMD of the cervical vertebrae.

The mean HU of Group VI was greater than that of Group V, but statistical analysis showed no significant difference between the two groups. We believe that the reason for this phenomenon may be that the decline in HU values reaches a cutoff value with increasing age. In addition, the sample size was small, thus creating a selection bias.

### Characteristics of the Distribution of HU Values in the Cervical Vertebrae

Previously, Ordway NR et al. (16) proved that there were no significant differences in HU values from C3 to C7 in eight cadavers. Anderst WJ et al. (17) studied 10 patients with cervical radiculopathy and 12 asymptomatic controls (aged 35-61) and found that C5 had the highest HU values in C3-C7. Zhang Y et al. (18) reported a cohort of 598 healthy adults between the ages of 20 and 64 years, with gradual decrease in HU values from C2 to C7, except for C3. Salzmann SN et al. (19) reported a cohort of 194 patients underwent ACDF with a mean age of 55.9, the mean HU value within C1 was 253.3, C2 was 276.6, C3 was 272.2, C4 was 283.5, C5 was 265.1, C6 was 235.3, C7 was 216.8, T1 was 184.4. In our study, we measured HU values from C3 to C7 vertebrae and found that C4 had the highest HU values among all subgroups, with a gradual decrease toward C3 and C7. We consider there are three possible explanations for the inconsistent results of these studies. First, the sample size may have had an impact on the final results. There were only 8 members in our Group I. Although the measured HU values were the highest in C4, the statistical differences between vertebrae were not significant. Similarly, the sample size in the study by Ordway NR et al. was 8 (16). Second, both the selection of the region of interest and the measurement methods were different. We measured the HU values of cancellous bone within the vertebral body, whereas Anderst WJ et al. divided the entire cervical vertebrae into 11 regions for measurement and calculated their mean values (17). In addition, ethnic may also be a reason. Salzmann SN et al. (19) had 91.2% Caucasian in their study, while Zhang Y et al. (18) and our study individuals were all Chinese.

To our knowledge, this study reveal the BMD characteristics of the cervical spine by separating the upper and lower parts of the vertebrae firstly. We found that in the cervical spine, the HU values within the vertebrae was not uniform, in particular, the differences were statistically significant between upper and lower parts of C6 and C7. In all our subgroups, the highest point of HU values was in the upper part of C4 and gradually decreased toward the upper part of C3 and the lower part of C7 (except for Group I, probably because of its small sample size). We consider two possible explanations for this feature. Firstly, the direction of force on each cervical vertebra is not vertically downward due to the presence of cervical lordosis. According to Wolff’s law, we know that the structure of the bone is affected by the direction of mechanical stimulation. In the sagittal position of the cervical spine, C4 tends to be located at the apex of the cervical lordosis, which is also the site of stress concentration. Therefore, the highest HU values are found in the C4 vertebrae. Secondly, the volume of the cervical vertebrae gradually increases from C3 to C7. Therefore, the pressure per unit volume is different in different vertebrae. In particularly, the C6 and C7 vertebrae are significantly larger and have a larger cross-sectional area (20). Therefore, the BMD of the C6 and C7 vertebrae is lower than that of the vertebrae above.

### Correlation of Cervical Vertebrae HU Value With the Intervertebral Disc Degeneration

Anatomically, the vertebrae and intervertebral discs combine into bundles to form the motor segments of the spine. Mechanically and biologically, they are closely linked and are considered to be a functional unit (21–23). The vertebral endplate is a thin layer of cartilage structure between the vertebrae and the intervertebral disc, which plays an important role in the nutrient supply and stress transmission to the disc (24). Intervertebral disc degeneration and osteoporosis are the most common degenerative diseases of the spine, and the two structural degenerative processes often occur together. However, the detailed relationship between them is not clear. Disc degeneration is the weakening of disc structure and function due to various causes, such as genetics, aging, malnutrition, trauma, high loading, etc (25). Previous studies have shown that inadequate nutrient supply to intervertebral disc cells is a major event in the initiation and progression of disc degeneration (26). The intervertebral disc is the largest non-vascular structure in the body, and the material exchange of the disc depends mainly on the nutrient pathways of the upper and lower endplates, and damage to the endplate nutrient pathways will cause disc degeneration (27–29). Therefore, the integrity of the endplate, which serves as a bridge between the vertebral body and the disc, may be a key factor in disc degeneration.

Osteoporosis can cause endplate thinning and microfractures, which will affect the healing of the injured endplate, further reducing the vascularity near the degenerating endplate and increasing endplate calcification. The increased endplate calcification and decreased vascularity will affect the material exchange of the intervertebral disc, further promoting disc degeneration (28, 30). In addition, endplate fracture produces abnormal stress distribution in the adjacent disc, increasing the risk of internal damage and degeneration (22). In the current study, the grade of cervical discs degeneration were negatively correlated with the HU values of the adjacent vertebrae, and the HU values of the vertebrae above and below the disc showed a tendency of decrease with the aggravation of the disc degeneration. This result suggests that reduced vertebral BMD and vertebral osteoporosis may trigger or exacerbate adjacent disc degeneration. Another feature of this study is the first demonstration that cervical disc degeneration is strongly correlated with vertebral BMD based on HU value measurements, which is superior to DEXA scans in determining osteoporosis.

Interestingly, cervical disc herniation occurs most frequently in C45 and C56, whereas our study showed a progressive decrease in HU values in C4-C7 vertebrae. This is because we considered the cervical spine as a whole and considered the effect of gravity. In contrast, the C45 and C56 discs were also subjected to lateral shear forces, which did not conflict with our results. When each disc was analyzed separately, the HU values of the upper and lower vertebrae of the disc remained on a decreasing trend with increasing disc degeneration (**Figures 4**, **5**).

## Limitations

There are some limitations to the current study. Firstly, we collected data on patients with degenerative cervical spine disease requiring hospitalization, and further investigation is needed to examine whether the same trend exists in the normal population. Secondly, the samples collected in our study were all Asian, and whether the results are the same for samples from other ethnic groups requires further investigation.

## Conclusions

HU values are not typically consistent throughout all levels of the cervical spine and the distribution within the vertebral body is not homogeneous. Decreased vertebral bone mass and vertebral osteoporosis may trigger or exacerbate the adjacent intervertebral disc degeneration.

## Data Availability Statement

The raw data supporting the conclusions of this article will be made available by the authors, without undue reservation.

## Ethics Statement

The studies involving human participants were reviewed and approved by Medical Ethics Committee of the Third Hospital of Hebei Medical University. Written informed consent for participation was not required for this study in accordance with the national legislation and the institutional requirements.

## Author Contributions

WD, HW, and XL contributed to conception and design of the study. XL and QL performed the literature search, acquired, and collated the data, which were analyzed by XL and JX. XL wrote the first draft of the manuscript. All authors contributed to manuscript revision, read, and approved the submitted version.

## Funding

This study is supported by Medical Research Program of Hebei, China (20190073).

## Conflict of Interest

The authors declare that the research was conducted in the absence of any commercial or financial relationships that could be construed as a potential conflict of interest.

## Publisher’s Note

All claims expressed in this article are solely those of the authors and do not necessarily represent those of their affiliated organizations, or those of the publisher, the editors and the reviewers. Any product that may be evaluated in this article, or claim that may be made by its manufacturer, is not guaranteed or endorsed by the publisher.
